# Information Resource Needs and Preference of Queensland General Practitioners on Complementary Medicines: Result of a Needs Assessment

**DOI:** 10.1155/2011/810908

**Published:** 2011-04-17

**Authors:** Tina Janamian, Peter O'Rourke, Stephen P. Myers, Heàther Eastwood

**Affiliations:** ^1^Discipline of General Practice, University of QLD 4029, Brisbane, Queensland, Australia; ^2^Queensland Institute of Medical Research, Brisbane, QLD 4101, Australia; ^3^NatMed-Research Unit, Research Cluster for Health and Wellbeing, Southern Cross University, Lismore, NSW 2480, Australia; ^4^Medical Education Services Australia (MESA), Notting Hill, VIC, Australia

## Abstract

*Objectives*. To explore in a cohort of Queensland (Qld) GPs' their attitudes to; knowledge about; and practice behaviour regarding complementary medicines (CMs), and to identify their perceptions of need for information resources on CMs. *Design*. A faxed self-administered survey to a random sample of 800 GPs in Qld. *Participants*. 463 completed surveys were returned, representing a 58% response rate. *Results*. The majority of GPs had a positive attitude about incorporating CMs in their clinical practice; however, only 12% perceived they had adequate knowledge to be able to advise patients about CMs. GPs most preferred evidence-based resources for receiving information on CMs (fact sheets, booklets, and journals) that contain clinical, pharmacological, and toxicological information. Most GPs perceived a need for an information resource on herbal medicines, vitamins, minerals, and trace elements, and nutritional supplements. *Conclusion*. GPs are open to integrating CMs into their clinical practice. They identify a current lack of knowledge coupled with a substantive level of interest to learn more. GPs perceive a high level of need for information resources on CMs. These resources should be developed and readily available to GPs to increase their knowledge about CMs and better equip them in communicating with patients about CMs use.

## 1. Introduction

In Australia, consecutive population surveys have indicated that complementary medicine (CM) is widely utilised by the Australian population with at least half using complementary medicines (CMs) and one fifth using complementary therapies (CTs) [1, 2]. Increasing consumer interest in, and use of, CM has impacted general practitioners (GPs) acceptance of CM on a global scale. Studies conducted in Australia [3–5], and overseas [6–8], have shown that GPs generally have a positive attitude towards CM and are open to integrating CM into their clinical practice. The most recent national Australian study indicates that 21% of GPs used various CM techniques in their practices and 75% referred to both medical and nonmedical CM practitioners [3]. 

GPs are increasingly expected to address issues associated with CM [4, 9, 10] which has led to high level of interest in GP training in this field [4–7]. There is currently limited knowledge about what Australian GPs need in CM information resources and the type of CM information resources they prefer to use in their practice. Identifying these needs will help educators, educational institutions, and other interested organisations (such as the Australian Medical Association, Royal Australian College of General Practitioners, Therapeutic Goods Administration) to respond more effectively. The aim of this study was to explore Queensland GPs attitudes towards CMs, knowledge of CMs, and practice in relation to CMs, and to identify Qld GPs' perceived level of need for information resources on CMs, and their preferred type of information resource on CMs to use in their clinical practice.

## 2. Methods

A random sample of 800 Queensland GPs was obtained from Australasian Medical Publishing Company. A five-page questionnaire was designed based on exploratory studies which included a comprehensive review of existing literature, market research of CM courses, focus groups with local GPs, and feedback from an expert advisory group consisting of experts in the area of general practice and CM. After pilot testing, the survey was faxed to the 800 GP's practices in 2003. Two follow-up reminders were made to nonresponders. 

In Australia, the Therapeutics Goods Administration (TGA), a Division of the Federal Department of Health and Aged Care, defines complementary medicine (CM) as therapies (systems and methods) and products (medicines, devices) which “complement” the body's own physiological mechanisms or other medical systems. Complementary therapies (CTs) include acupuncture, chiropractic, meditation and massage and cover Eastern systems of medicine, Western systems of complementary medicine, ingestive delivery methods, manual delivery methods, mental/emotional/spiritual methods. Complementary medicines (CMs) cover products, such as herbal medicines, vitamins, minerals, trace elements, nutritional supplements, homeopathic and aromatherapy products [11]. Currently, the TGA definition for CM is the preferred term amongst most GPs and researchers in Australia [12]. 

As the area of CM is so diverse, it was decided to limit the CM modalities in this study to CMs and not CTs. Therefore, the TGA definition of CMs was used. This decision was confirmed based on the findings of the exploratory study including a literature review which indicated that consumers are commonly self-medicating with CMs such as herbal medicines and vitamins and mineral supplements [1, 2, 13] and focus groups findings which indicated that GPs perceived a need for information resource on CMs rather than CTs. The following CMs were included in the survey: herbal medicines, vitamins, minerals and trace element, nutritional supplements, dietary interventions, homeopathic medicines, and aromatherapy products. 

The five-page needs assessment survey contains 22 questions which are divided into five major components: (1) current perceptions about CMs, (2) current knowledge about CMs, (3) current clinical practice of CMs, (4) information resource needs on CMs, and (5) demographics. Once the completed questionnaires were received they were coded for data entry and analysed using SPSS (Version 10.0). Statistical analysis included chi-squared tests, *t*-tests, logistic regression and general linear modelling to examine the strength of association between variables. 

## 3. Results

### 3.1. Demographic Characteristics

The overall response rate for returned surveys by GPs was 58% (*n* = 463). The representative nature of the survey respondents was compared using the General Practice Workforce 1999 data from the Australian Commonwealth Department of Health and Aged Care [14] and the Bettering the Evaluation And Care of Health (BEACH) survey of general practice activity 1998–2003 [15]. The survey respondents were slightly overrepresentative of female and younger GPs (<35 age group). The respondents were underrepresentative of GPs in the “capital city” and overrepresentative of GPs in the “other metropolitan” areas (Table 1).

### 3.2. GPs' Attitudes towards CMs

Almost all respondents (96%) perceived that their patients are using CMs. Half of responding GPs considered CMs are useful supplements to regular medicine. The majority of GPs' perceived that they have an ethical responsibility to ask their patients about their CMs use (74%) and to discuss with their patients scientifically proven CMs relevant to their care (65%). Even though most GPs (82%) perceived that they should have some knowledge about the most important CMs and they should be able to advise patients about CMs (54%), only 12% perceived that they had adequate knowledge to be able to advise patients about CMs. The majority (70%) of GPs perceived that CMs are not well regulated in Australia and 25% were unsure.

### 3.3. GPs' Knowledge of CMs and Interest for Further Education

Overall, 27 GPs (6%) had obtained or were obtaining a formal qualification / training on CMs, approximately half of respondents had used an information resource on CMs in the past 12 months, and 11% (*n* = 52) had attended a continuing medical education (CME) on CMs in the past 12 months.  Table 2 represents GPs' perceived level of knowledge of the different CMs modalities. Almost all responding GPs (97%) perceived they had “no” or “limited” level of knowledge of homeopathic preparations and aromatherapy products, and a high percentage of GPs (87%) indicated having “no” or “limited” knowledge of herbal medicines. Approximately half of responding GPs perceived a “moderate” level of knowledge of dietary interventions (55%), vitamins, minerals and trace elements (53%), and nutritional supplements (42%). A very small percentage of GPs perceived they had extensive knowledge of any of the six modalities investigated. 

Seventy-six GPs (16%) indicated that they were interested in undertaking formal education and 52% of GPs indicated interest in attending CME on CMs in the future. GPs reported being mainly interested in learning about herbal medicines, nutrition, and other commonly used CMs. The binary logistic regression model demonstrated that GPs' interest to undertake CME on CMs is associated with many explanatory variables such as GPs' positive attitude towards CMs, current knowledge of CMs, and GPs clinical Practice of CMs.

### 3.4. GPs' Practice of CMs

Table 3 includes GPs clinical practice behaviours with regards to CMs. Almost half of respondents indicated that they question patients about CMs, and 40% discuss safety issues about CMs, and record patients' use of CMs frequently. Nearly half of respondents reported that they prescribe/recommend CMs to their patients occasionally-to-frequently. Almost 20% of GPs practise use some CMs in their practice seldom-to-frequently, and almost 60% refer patients to medically qualified practitioners seldom-to-frequently, and 35% refer to nonmedical complementary therapists. Only 36% of GPs agree that they should get to know CM practitioners in their area and another 40% were unsure.

### 3.5. GPs' Level of Perceived Need for CMs Information Resources, Their Preferred Type of Information and Preferred Type of Resource

GPs' perceived the highest levels of need for an information resource on vitamins, minerals and trace elements (93%), herbal medicines (90%), nutritional supplements (90%), and dietary interventions (88%) (Table 4).

GPs' most preferred type of information to be included in a CMs information resource was evidence-based medicine information. The majority of GPs also wanted pharmacological, toxicological and clinical protocols on all of the six CMs modalities.

GPs ranked fact sheets, booklet, journal, computer-based, and workshops as their five most preferred resources for receiving information on CMs (Table 5). The least preferred types of information resources were telephone hot line and long seminars.

## 4. Discussion

This study was consistent with previous studies [3–5, 16, 17] and demonstrated that Qld GPs have a positive attitude towards being involved with CMs in their clinical practice. This was confirmed by their positive attitude towards communicating to patients about their CMs use and their incorporation of CMs into their clinical practice. Many GPs are recommending CMs as part of treatments, practising CMs modalities or referring patients for CMs treatments.

### 4.1. GPs' Lack of Education and Information on CMs

Our results indicate that only a small number of GPs perceived that they had adequate knowledge of CMs to be able to advise patients, a finding that is supported by previous literature [8, 18]. Although it was encouraging that half of responding GPs perceived having a moderate knowledge of dietary interventions, vitamins, minerals and trace elements, and nutritional supplements, given that only 6% of GPs had undertaken or were undertaking formal education on CMs, it is likely that the source of knowledge for those perceiving a moderate knowledge was mostly from CME courses, journals, and other resources such as the internet, patient, and drug companies. It was not possible to assess the extent to which this knowledge was evidence based.

The majority of GPs believed that they had none or limited knowledge of herbal medicines, aromatherapy products, homeopathic preparations. It is expected that many GPs would have no knowledge of homeopathy or aromatherapy as these modalities are not as commonly used by the Australian population, thus GPs' exposure to these modalities would have been minimal, if any. However, it is a concern that so few GPs perceived no or limited knowledge of herbal medicines when half the Australian population are using them [1, 2] and would increasingly turn to their GPs for advice. Other studies have reported that Australian GPs appear to know more about nonmedicinal modalities such as acupuncture, hypnosis, meditation, and chiropractic, and to a lesser extent about herbal medicines and vitamin and mineral therapy [4, 5]. GPs' inadequate levels of knowledge of CMs, in particular herbal medicines, is likely to negatively impact on their communication with patients about these modalities and as a result may compromise patient safety. Specifically, poor knowledge about herbal medicines which are in common use by the population has the potential to cause more dangerous side effects and interactions with pharmaceutical drugs.

### 4.2. GPs Interest and Need for CMs Education and Information Resources

Similar to previous studies [3–5, 7, 8], many Qld GPs indicated considerable interest to learn about CMs through CME and formal education. Studies have reported that GPs' main reasons for wanting to learn more about CMs is to be able to advise patients about CM and recommend safe and effective CM or to dissuade patients of unsafe CM [7, 19]. Likewise, GPs in this study were aware of patient demand for CMs with almost all GPs agreeing that their patients are using CMs and they perceived an ethical responsibility to ask patients about their CMs use and to discuss with their patients scientifically proven CMs. The AMA [20] and RACGP/AIMA [21] Position Statements have also stressed that “medical practitioners should specifically ask patients about their use of CM and take account of this in their management of conditions. Medical practitioners should be sufficiently informed about CM to be able to provide advice to patients when appropriate.” Therefore, it was an expected finding of the study that almost all responding GPs perceived a high level of need for an information resource in different CMs modalities, in particular for herbal medicines, vitamins, minerals and trace elements, nutritional supplements, and dietary interventions. Similarly, other studies assessing information needs of GPs in Canada and the US have found a high level of interest and need for information on CMs [22–24]. 

The majority of Qld GPs wanted the CMs information resource to contain evidence-based literature and pharmacological information. This is not surprising, given that the traditional evidence hierarchy is an important part of the evidence-based medical model. Suter et al. [24] reported that Canadian GPs also had a strong preference for evidence-based information such as systematic reviews and randomised controlled trials on CM. Similarly, other studies [18, 25] have reported GPs positive attitude towards evidence-based medicine information on CM. Overall, Qld GPs, like GPs in Alberta [24], prefer printed material such as fact sheets, booklets, and journals for receiving information on CMs. Dooley et al. reported that Australian oncology practitioners were found to mostly use paper-based materials such as textbooks and journals on CM [26]. Other healthcare professionals, such as pharmacists, have also reported that their primary sources of information on CM are books, magazines and journals [27–29]. Jackson and Kanmaz conducted an overview of information resources for herbal medicinals and dietary supplements and concluded that healthcare professionals often use book and compendia as their first resource when faced with questions about CM [30].

### 4.3. Need for More Regulation of CMs and CMs Practitioners

It should be noted that GPs attitude towards involvement with complementary practitioners was less enthusiastic with only a third perceiving that they know or should get to know nonmedical complementary practitioners in their area and 40% were uncertain. GPs' reluctance towards involvement with CMs practitioners was also confirmed in this study with more Qld GPs referring to medically-qualified complementary practitioners than nonmedically qualified complementary practitioners. GPs reluctant attitude about being involved with CMs practitioners may be due to their lack of knowledge of CMs practitioners' professional and educational qualifications and the perception that there is a lack of regulation of CMs practitioners in Australia. This notion is supported by another significant finding of this study which showed that 70% of GPs believed that CMs are not well regulated in Australia and 25% were uncertain. Cohen et al. also found that Australian GPs felt that most CMs need to be regulated [3]. Hall and Giles-Corti found that GPs in Western Australia who were against referral of patients for CM stated lack of government regulation and training standards as the reason for their view [4]. The AMA position statement on CM, states that “it is essential that there is appropriate regulation of complementary therapists. Such regulation should ensure that nonmedical complementary therapists cannot claim expertise in medical diagnosis and treatment” [20]. This study supports the AMA Position Statement about the importance of CM and CM practitioner regulation as it appears to be a concern for GPs and is likely to influence their attitude and practice of CMs. Improvement of CM regulations in Australia is likely to increase the confidence of GPs to refer to and communicate with CM practitioners and patients more openly about CM.

### 4.4. Limitations

This study had several limitations. When comparing the characteristics of respondents to the GP population in Australia and Qld, there appeared to be underrepresentation of male and older GPs. This is not surprising considering that female and younger GPs have been found to be more interested in CMs, therefore more likely to respond to the questionnaire. The study instrument was a self-report needs assessment survey so the validity of self-reporting, recall bias, and response rate needs consideration in interpreting the findings. It is also important to mention that the needs assessment survey used in this study was a subjective assessment of GPs' needs, and subjective need does not necessarily equate to actual need [31]. 

## 5. Conclusion

In summary, Qld GPs are favourable to incorporate CMs into their clinical practice; however, their current level of education and knowledge of CMs does not allow them to do this adequately. GPs willingness to learn more about CMs and their high level of need for information resources on CMs suggests that more opportunities should be provided to GPs to increase their knowledge of CMs. This study provides unique data on Qld GPs information resource needs on CMs which can assist in developing appropriate resources on CMs for GPs to use in their clinical practice.

## Figures and Tables

**Table 1:** Comparison of the Australian and Queensland GP population with the study respondents.

GP demographics	GP population [14] (*n* = 18,787)	Qld sample [15] (*n* = 933)	Respondents (*n* = 463)
Gender			
Male	66%	64.6%	62%
Female	34%	35.4%	38%
Age distribution			
<34	12%	7.1%	17%
35–44	32%	33.9%	36%
45–54	30%	33.2%	29%
55+	26%	25.8%	17%
Rurality			
Capital city	68%	50.6%	41%
Other metropolitan	7%	14.1%	32%
Rural/remote	25%	35.4%	27%

**Table 2:** GPs' perceived level of knowledge of six different CMs modalities on a 4-point-scale of “no knowledge” to “extensive knowledge” (*n* = 463).

Perceived level of knowledge				
CMs modalities	No knowledge %	Limited knowledge %	Moderate knowledge %	Extensive knowledge %
Herbal medicines	17	70	13	0.2
Vitamins, minerals and trace elements	4	37	53	6
Nutritional supplements	7	47	42	5
Homeopathic preparations	62	35	3	0.4
Aromatherapy products	62	35	3	0
Dietary Interventions	6	31	55	8

**Table 3:** GPs' clinical practice of CMs (*n* = 463).

Statement	Never	Seldom	Occasionally	Frequently
	%	Few times a year %	Few times a month %	At least weekly %
I question my patients about their complementary medicines usage	2	17	36	44
I discuss safety issues about complementary medicines with my patients	2	16	42	40
I record patients' use of complementary medicines in their medical file	4	20	38	38
I prescribe/recommend some complementary medicines to my patients (e.g., nutritional supplements, herbal medicines)	18	32	33	17
I practise some complementary medicines in my practice (e.g., homeopathy, aromatherapy)	81	10	5	4
I refer patients to medically-qualified complementary practitioners	42	37	15	6
I refer patients to nonmedically qualified complementary therapists	64	23	9	4

**Table 4:** GPs level of need for an information resource on different CMs on a 4- point-scale from “no” need to “high” need (*n* = 463).

CMs modalities	Level of need				
	No %	Low %	Moderate %	High %	Some %
Herbal medicines	10	31	47	13	**90 **
Vitamins, minerals, trace elements	7	35	46	13	**93**
Nutritional supplements	10	34	46	11	**90**
Homeopathic preparations	45	36	15	4	**55**
Aromatherapy products	50	37	10	4	**50**
Dietary interventions	12	27	43	18	**88**

**Table 5:** Cumulative percentages of GPs' top five preferred type of information resources for receiving information on CMs (*n* = 463).

Type of information resource	Top one %	Top five %
Fact sheets	26	85
Booklet	17	76
Journal	16	63
Workshops	9	54
Computer based	11	52
Short seminars	5	42
Book	8	37
Web page	5	34
Telephone hot line	0.6	18
Long seminars	0.4	7
